# Selected Cases, with Medical and Therapeutical Observations

**Published:** 1860-12

**Authors:** F. Peyre Porcher

**Affiliations:** Physician to the Marine Hospital, Charleston, S. C.


### SELECTIONS.

[From The Charleston Medical Journal & Review.]

### *Selected cases, with Medical and Therapeutical Observations.*

By. F. Peyre Porcher, M. D., Physician to the Marine Hospital, Charleston, S. C.

1. Removal of "Horny Tumor" of tne second growth, and of uuusual size.—2. Imperforate Lachrimal Duct dilated by Membranes.—3. Scrofulous Degeneration of Bones with Oxalate of Lime in the Renal Excretion.—4. Relation between the Accumulation of Fat in the System and Atrophy of the lungs.—5. Abortive Treatment of Blennorrhagia.—6. Influeuce of Chloroform in the Recuperation of the System during Protracted Labors.—7. Hoffman's Anodyne a Substitute for Alcohol and Opium in Delirium Tremens.—8. Isolated Epidemic of Varioloid in Charleston.—9. Large doses of Opium in Centric Convulsions of Children.—10. Operations for Relief of Excessive Seminal Losses simplified.—11. Traumatic Tetanus successfully Treated.—12. "Irrigation" in Farctures; and treatment of Ulcers by applications of Water at High Temperatures.

*Case of a "Horn" of Extraordinary Size and of the Second Growth, removed from the Head of a Negro Woman.*—The readers of this Journal, and those who saw the patient, will remember a report made in the March number, 1855, of a case in which a horn some seven inches in length was removed from the head of a negro woman. The history of the case was accompanied with a notice of such growths, and a wood-cut representing the deformity. In its removal a portion of the cartilaginous base remained, and the horn re-appeared, attaining such a size (three inches on one border and two-and-a-half on the other, with a diameter at its base of two inches, these dimensions being themselves extraordinary,) as to require a second operation for its excisions. The interval between the two operations was twenty-four months, by which, of course, we have an indication of the rate of growth of such horny tumours.

Chloroform being administered to the patient, in the presence of Prof. E. Geddings and Drs. Cain and Chisolm, and the class of the Medical College of the State of South Carolina, it was completely dissected away, Dec. 1856. No portion of its cartilaginous base was allowed to remain, and the portion of the cranium on the left side of the head at the junction of the parietal and frontal bones, constituting the base upon which the tumor rested, but to which it was in no wise attached, was completely exposed. The wound healed, and there has been no return of the disease, nor indeed any further possibility, it would seem, of such re-appearance.

*Case of Imperforate Lachrimal Duct, treated by Dilatation of Animal Membranes for this purpose.*—Mrs. ——, of Savannah, Ga., consulted me for an aggravated, long-standing and inconvenient discharge of tears, with a dryness of the right nostrial. There was no Fistula Lachrimalis. Upon using the fine and sharp silver probes adapted to the purpose, I found the channel through the lachrimal canal to the nose impervious on that side. After persevering some two weeks in the daily injection, with Anel's syringe, of a solution of sulphate of copper in the endeavor to force a passage, but without effect, I attempted gradual dilation with silver probes introduced into the lachrimal sac through the superior punctum lachrimalis. After much persevering effort, making slow progress each day, I at last succeeded in passing the dilating probe into the noseril. With the asssistance of Dr. T. L. Ogier, it was drawn through the nose from the eye, having a bit of silk thread passed through the eye of the probe, the other extremity of the thread being previously lashed to a string of cat-gut of small size. The latter was in this way drawn through to the nose and worn for several days. By the absorption of fluid it dilated the passage. A larger sized cat-gut, carefully joined to the extremity of the first used, in like fashion made to occupy its place. It was worn for several days before being removed; and thus, by absorption and consequent swelling of the animal fibre, complete dilatation of the paasage to the nose was affected. The patient was, as might have been expected, much benefited. I have not seen her for some time past and cannot state what has been the permanent result. The case is reported to show to what extent, by persevering and gradual effort, a completely occluded passage can be re-opened without the use of the knife; and what a large sized thread can be worn without material suffering in these seemingly sensitive channels. In true fistula lachrimalis the use of the knife would undoubtedly be required.

*Case of Generul Scrofulous Degeneration of the Bones, coëxistent with the escape of Oxalate of Lime from the Kidney—soft tissues remaining unaffected. Important diagnostic mark deduced.*—After some experience in the use of the microscope and the application of chemical tests and reagents, I have been struck with the comparative rarity with which crystals of oxalate of lime are met with in this city. The body of a man, æt. 30, in the Marine Hóspital, was so marked with cicatrices that the diagnosis of a casual or inattentive observer would undoubtedly have been *scrofula* affecting the soft tissues; but these, upon closer inspection, proved to be upon those parts of the surface where bones or portions of bone, after suppuration, had found an exit from the system—the soft tissues readily healing, and there being no glandular degeneration whatever. The general appearahce of the patient, his functions, appetite, nutrition, etc., were good. Aware, however, that my predecessor had previously removed portions of these bones by excision, and seeing others also in process of separation and discharge, I approached the patient with the confident expectation of finding oxalate of lime in the renal excretion; the kidney, by a wise process of nature, being the emunctory for the removal of these products, disintegrated, thrown off, and if not removed liable to prove injurious to the system, and generally detrimental to health; as is, under analogous circumstances, the accumulation of sugar, albumen, urea, the phosphates, etc. The repeated examinations revealed oxalate of lime as the characteristic element, and in large quantity.

Independently of the comparitive rarity of the crystals of oxolate of lime in the urine, in this city, so far as my observations extend, this case is interesting for two reasons: First, it shows the connection between scrofulous degeneration of *bone* and oxalate of *lime* in the urine. Secondly, it conflicts with the opinion expressed by Golding Bird, and generally received by other writers on Urinary Pathology, that the escape of oxolate of lime in excess (oxaluria) is *characteristic* of injury to the nervous system, or that it indicates loss of cerebral or brain matter. This is the more strange, as the *phosphates* predominate in the composition of nerve substance, but phosphates, together with *lime*, in the composition of bone.

Notwithstanding this, in his entire chapter referring to the Oxalate of Lime in the Urine, Dr. Bird (upon whose work I set a high value as a manual in English, though Robin says "*Il est beaucoup copiées*,") does not once consider the lime element in the destruction and separation by the kidney of disintegrated bone; nor does he allude to its connection with disease of the bony system. The nervous system in this individual was not in the slightest degree affected, notwithstanding the great amount of oxalate of lime excreted. His intellect remained perfectly clear, and his nerves unshaken, even when he became dropsical from the waste going on and consequent impairment of the blood; and when albumen as well as sugar was escaping with the urine, both of the latter products being also found, one of them by Moore's test, in the fluid drawn at a later period from the cellular tissue by the trochar.

My experience amply confirms, on the other hand, the truth of the coëxistence of the triple phosphates in *excess* with derangement and disease of the *nervous system*; invariably too, both from the number and size of the crystals.

It may be observed, from my note on the Hospital Book, upon a third examination, Oct., 14th, that the bones had ceased to suppurate, the octahedra of lime completely disappearing from the urine, with symptoms of general dropsy supervening. The intellect continued perfectly undisturbed, there being no nervous disturbance up to the time of his death.

*Accumulation of Fat in the System, dependent upon Atrophy of the Lungs, explicable upon the Chemico-Physiological theory of Liebig and Draper on Combustion.*—This connection is best illustrated by the details of a case with the post-mortem. During my attendance, for some eight months in 1851, at the Charleston Alms House, Green, a man of plethoric habit, full and large of person, but intemperate, became an inmate of the hospital. His breathing was difficult and labored, there was extensive dullness upon percussion of the thorax and almost complete absence of respiratory murmur; otherwise the patient seemed fleshy, strong and robust.—Death ensued in a few days without emaciation, and with no depletive or weakening treatment whatever having been put in practice. The notes of the autopsy extracted from my case book reveal and explain his hopeless condition, and at the same time furnish a most plausible explanation of his *apparent* promise of life:

Green, æt. 39, had been occasionally attacked with delirium tremens. There was revealed enlarged liver of a cinnamon color; consistence firm; gall-duct full; no yellowness of conjunctivæ. (In another post-mortem, where the patient had been taking calomel for two weeks I yet found the gall-duct full of bile.) Stomach injected, empty; veins very large; pleural cavity of right side *filled with fluid*, to which it was confined, producing almost complete atrophy of right lung, and accounting for the dullness and the entire absence of respiratory murmur. Some two quarts of fluid were found in the cavity of the thorax. The left lung, dark and crepitant solely in the upper lobe, was partly infiltrated with serum, and *in it alone was respiration carried on. Heart fatty and containing fibrinous deposits.* Person muscular and corpulent; large accumulation of fat, accounted for by the inability of the lungs to decarbonize the blood. Brain natural, save some injection of pia-mater. Patient up to his death was in his right mind, and had not complained of headache. Opium was administered the day previous to his death to relieve pain. *Mesentery and omentum fatty.* Treated by leeches, small doses of calomel during one day, blisters, cupping, poultices, etc.

APPLICATION.—From the almost complete atrophy of both lungs there was consequently no *combustion* of the fat in the system; the carbon introduced by food, etc., as well as that usually eliminated as a product of the waste of tissue by respiration in the shape of carbonic acid gas, accumulated in the system; for the hydrocarburets consumed, together with his free potations of alcohol, failing, from the deficiency of oxygen, to be eliminated by the respiratory process, in proportion as they accumulated in the blood, were deposited in the integuments. There was consequently a thick coating of fat over the abdomen, on the thighs, and between the layers of cellular tissue, giving him the appearance of one who would be called, at a casual view, a healthy man.

The analogous facts regarding the rapidity and slowness respectively of the circulation in arctic and tropical countries, is suggestive of useful comparison; There is a necessity in those residing in the first for highly carbonized and nitrogenous food; in the other, from the diminished respiration, ascescent vegetables and abstinence from carbonaceous food and alcoholic drinks is demanded, and there is danger resulting consequently from congestion and fatty degeneration of the liver, in tnose indulging too freely.

*Abartive Treatment of Gonorrhœa, with Tartar Emetic; cure in thirty-six hours.*—In an attendance for several months, some years since, upon the clinical lectures of Prof. ———, at the College of Physicians and Surgeons, ot New York, my attention was called to the pathology and treatment of gonorrhœa and its relations to diseases of the mucus surfaces. If antiphlogistics, expectorants, tartar emetic, etc., are active in lessening inflammation and in inducing secretions in the mucus surfaces of the lungs, so are they effectual in reducing inflammation seated in other analogous structures with different functions, but where inflammation can be cut short just as certainly. By the bold use of a term the professor asked why we could not give as it were an expectorant in gonorrhœa also, at the same time suggesting the use in its forming stage of so powerful an antiphlogistic as tartar emetic. I have repeatedly availed myself of this hint, and with almost uniform success.

Method.—In the formative stage of the disease, soon after its appearance—within the first fifteen to thirty-six hours, if possible—and before the inflammation has made any advance, order the recumbent posture to be preserved, and the use of warm mucilaginous drinks with nitre and doses of tartar emetic sufficient to keep up constant nausea. The sedative circulates to the remote parts of the system, the progress of the inflammation is thus put an end to, and in my experience, with but one exception, the disease completely arrested. It is desirable that more extended trials should be made. As we might naturally anticipate, under the powerful depressing and sedative influence of the antimonial upon the circulation, inflammation can make no progress; the urine at the same time is diluted by the mucilaginous drinks; rest and the recumbent posture all contribute their due influence to prevent the march of a disease, leading on when unchecked to acute inflammation, congestion of the urethral mucus membrane and, ultimately, suppuration with protracted and exhausting discharges. The addition of a very weak injection of sulph. of zinc, or other astringent, I have also found not injurious, and possibly it may contribute to change the growing tendency to morbid action in the parts. Any objections urged against the severity of the means recommended may be met by the consideration of the tedious and protracted character of the treatment ordinarily used, the sum of inconvenience being much in favor of the nauseant. The patient should of course report himself at the earliest possible moment, as the success obviously depends upon the little progress made by the inflammation.

Dr. Billing ("Principles of Medicine") treats diseases of the skin, as well as the affections where minute capillaries and absorbents are concerned, with tartar emetic, iodide of potash, etc., which he contends circulate to and thus modify the parts locally. Thus affections apparently most opposite in character, as for example, pneumonia, mentagra, inflammation of the absorbent glands from wounds and poisons, vesicular disease of the lungs, etc., are relieved, there being both lessening of the impulsive force of the heart and the local effect referred to.

*Employment of Chloroform in protracted Obstetrical Cases, not primarily to relieve pain, but to produce sleep during the intervals of the pains.*—Amidst all the discussions respecting the policy or not of employing chloroform in midwifery, I have been struck with the practical advantage of administering it (a few drops inhaled from a handkerchief) in the *intervals* of the pains of tedious labors to *cause sleep.* It is during this sleep, which oftentimes only a sedative like chloroform will afford, without injury to the patient, that her exhausted system recovers from the effects of protracted suffering. In tedious cases, among primipara particularly, where labor is protracted sometimes during two or three days and nights, there is much exhaustion, not alone from pain, but from the absolute want of sleep. In these cases where it is impossible from the nature of things to use any other remedy (as for example Graves', of tartar emetic and laudanum in the insomnia of typhoid fever,) I have applied chloroform as directed above; or gave it to the attendant to be employed in this cautious and safe way, during my absence. Sound and refreshing sleep even of a few minutes between the pains was thus secured; and I have observed parturient females in a better condition at the end of the fortieth hour than they had been, when not using chloroform, at the conclusion of the first day of labor. Those who have once obtained relief in this way are not likely to decline the aid of the drug. The anæsthetic thus used does not seem to interfere with the progress of the case, and is really not forbidden by a single good reason—the usual objections to the employment of chloroform in labor not applying to this mode of using it. It has been employed by me simultaneously in those cases also requiring the use of ergot, as the following will serve to illustrate:

June 11th, 1857, called to see Mrs. F., primipara, æt. 21. Pains had commenced at 3 o'clock in the morning; I saw her at 2 p. m., found head presenting and resting on symphisis pubis where it remained that day and night, also the next day until 8.30 p. m., when further progression down wards commenced. No swelling of the bag of waters occurred until the last mentioned period; it was ruptured just before delivery, which was effected at 12 p. m. of the second night, the labor being thus near forty-eight hours in duration. The os uteri was soft, relaxed and dilated from my first examination. After the first day I found chloroform very useful repeatedly employed; it gave sleep between the pains, the severity of which it also lessened, thus being a useful *restorative;* so much so that at the conclusion of the second day of her labor the patient was in every respect in a more favorable condition than during the first twenty-four hours. I also gave her ʒ ii of the powdered substance after taking ℥ i of the tinct. of ergot in divided doses every half hour, which acted effectually. In this case, as in others in which I have used it, the uterine action followed in ten to fifteen minutes after its administration. In a patient whose case I reported in the Journal, and where the labor was protracted to the seventy-eighth hour, I am sure that the use of chloroform after the method mentioned above would have lessened the amount of suffering endured, and which proceeded principally from continued loss of rest.

*Hoffman's Anodyne, a Substitute for Brandy and Alcohol in the Treatment of Delirium Tremens.*—Seeing much of this disease in the wards of the Alms House Hospital, during my attendance there some years since (1851), and having noticed the good effects of the comp. spirit of sulphuric ether in most cases of nervous excitement and exalted nervous sensibility in females, I determined to administer it in an analogous condition, caused by excessive potations of alcohol whether occurring among males or females—at the same time diminishing materially or entirely the usual amount of brandy or other stimulants so universally prescribed.

It was used with satisfactory results in eight cases, one drachm being administered diluted with water repeatedly until nervous tremor was allayed and sleep induced. I regret not having an opportunity for testing it further, but publish the above, without extracting the history of each from the Hospital book, in order that its value may be fully ascertained. Hoffman's anodyne would no doubt be a useful addition to the ordinary prescriptions containing opium as it possesses both a stimulant and antispasmodic action. I have also found Graves' combination for the insomnia of typhoid fever most efficient when the same condition exists in delirium tremens: ℞. Tr. Opi. gtt. xl, T. Emetic gr. ¼, repeated every half hour till sleep is induced. I have seen it, in patients in the Marine Hospital, completely effectual in producing sleep, for the want of which, during a period sometimes extending over four days and nights, the patient was in iminent danger.

*Epidemic of Varioloid appearing in and confined to a single building in the city of Charleston.*—It fell to my lot as physician to the Church Home, Laurens street, to note the origin and history of an epidemic of varioloid which suddenly and mysteriously appeared there, and affected 22 of the 32 inmates of the Institution—the disease being confined to and not spreading out of the House. Non-intercourse was enjoined, the beds and bedding were destroyed, purification and painting of the house followed its disappearance, and no death occurred. Several who were vaccinated took the disease, even those too who had been just vaccinated and with fully formed pustules maturing; but the severity of the symptoms seemed in every instance to depend upon the previous vaccination or its absence. The cases presented every grade of intensity from the slightest irritative fever with a few scattered eruptions and pustules, to others the surface of whose bodies were encrusted with a mass of disease, with inflammations and swelling of the cellular tissue over the entire body, so that the eyes were not visible. In two cases this was followed by troublesome suppuration. The treatment was exceedingly simple, consisting of local applications of olive oil, of a laxative on the first appearance of the disease followed by mucilaginous drinks, and as a diuretic a weak solution of chlorate or citrate of Potash with a little camphorated Tinct. of Opium. But one sufferer was severely pitted. The ages of those affected varied from 2 to 45 years. The source of this dreaded disease could not be traced, though two cases of small pox did occur in a remote portion of the city, and the disease was said to exist at the same time in Georgetown, S. C., where the mother of one of the girls resided. The latter however was exempt. The strictly isolated character of the epidemic and its unprovoked appearance conslitute its chief points of interest.

*Time of appearance and disappearance of Varioloid.*—(From my note book.) "The first case of Varioloid at the Church Home occurred the last week in February. There were seven by the first week in March; eruptions out on the 3d, 4th and 7th day; on the 8th full on the face; on the 10th they appeared on lower extremities, commencing to dry up from the 9th to 12th. The disease commenced to disappear about April 1st, though as late as the 20th of April there were large suppurating ulcers requiring opening under the skin of head and face of one little sufferer who was pitted." A man with small pox died under my care in the Marine Hospital in 1858; only 3 out of 25 inmates took the disease; these were transferred to the pest house and recovered. The infection was thus timely extinguished.

*Use of large doses of Opium for the relief of Centric Convulsions in children, not dependent upon worms, errors of diet, etc. Value of the reasoning derived from the action of therapeutic agents considered.*—With sufficient defference to those extremists who reject the idea of any benefit being dependent upon the medicines employed, or that any useful results flow from judicious treatment, however cautiously employed, I claim that the reasoning most fruitful in result *is often* that which is *a posteriori*, and derived from an attentive consideration of the effect of our therapeutical applications. The repeated or unvarying success following the judicious use of certain remedies, possessing a special mode of action, may by the attentive observer be made to reflect light upon, and give a clue to, the nature of the disease itself.

For example, in the opposite conditions of spasm and relaxation, the uniform relief following the employment of an agent, the action of which is well known, enables us to draw conclusions as to the cause of the symptoms, if it does not teach the ultimate nature of the malady; besides the practical hint it conveys concerning the useful repetition of the same means in other cases of the like character—Tartar emetic producing the well known effect, viz: nausea, relaxation and consequent relief in cases of spasmodic colic, whether it be administered by the mouth or thrown into the rectum, confirms our theoretical opinion respecting the presence of rigidity and muscular contraction affecting the intestine. The same is true with respect to its use in pneumonia or in hernia, by relaxing the air cells in the one case or the constricted intestine in the other; or if we are inclined to agree with Billing, that all purgatives, emetics, etc., are sedatives, and when circulated through the system that they contract the capillaries: still our reasoning whether to sustain the one doctrine or refute the other is based in part upon a direct reference to the action of the agent employed. To select another most simple and obvious illustration: the alkalies, soda, chalk and other absorbents, in relieving acidity and flatulence, *prove* more certainly still that the cause of the painful condition consists in the presence of *acid* accumulations, vitiated secretions, gasses, etc.; for these are modified when the agents above referred to are brought into contact with them out of the body, in the laboratory of the chemist for example.

When Dr. Meigs ascertained that by turning an infant affected with cyanosis on the right side, and thus by lessening the tendency to the flow of blood in the valve of Bottali in the new born with defective closure of it, he was not merely enabled to relieve the sufferer, but to reason back and assert the special condition of the circulatory apparatus. In cases, too, where, notwithstanding temporary relief, the patient died, the post-mortem served conclusively to confirm what the temporary relief afforded by the treatment had led him to suspect. Again, among diuretics, the constant relief following the use of acids or alkalies, respectively in the two conditions of acid or alkaline deposits in injurious excess in the urine, enable us to suspect or to know (by finding our suspicions confirmed by subsequent developments) that the kidney is diseased, or that the blood contains these products in hurtful amounts. The same is true of albumen, or sugar, in its indication of deranged integrity in the manufacturing process of the liver; hence the truth of the Hippocratic maxim I have sometimes quoted; "*naturam* morborum *curationes* ostendunt." I have been led by a similar process, namely, success obviously following the active means used, to draw important conclusions respecting the intimate nature of the disease; as in an instance like the following, where the use of a relatively large dose of opium completely put an end to a very severe and protracted form of convulsion in an infant. No other appreciable effect whatever, neither purgation nor vomiting, ensued upon the employment of the other remedies which had been previously employed. But with the induction of *narcotism* entire cessation of the most violent spasmodic movements, which had been protracted during three hours, ensued; which, had they continued, must have put an end to the life of the patient. Hence, conclusion: no inflammation as cause, no worms, or other material agency—nothing save nervous excitability or derangement of the nervous centers originating the convulsive movements as the essence of the disease. The experience is practical. The details of the sase, taken at the time, are as follows:

"Called to a child, æt. 2, belonging to Mr. W.; found him in violent convulsions, which lasted three hours—no cause obvious—had eaten only a little food, unirritating in character—not teething—no worms—no eruptic disease—could not induce vomiting by ippecacuanha or sulph. of zinc—used terpentine enamata with oil—sinapisms produced no impression on skin, either any redness or pain. In last extremity gave enamata of 30 drops tinct. of opium in two ounces of mucilage—patient gradually recovered but remaining narcotized for three hours, with cessation of convulsive movement—increased frequency of pulse, fever, heat of skin and *contracted pupils* (they had been widely dilated at the beginning of the attack); cold was also applied to the head. It would appear that chloroform and laudanum might prove a useful combination in these cases which are not dependent upon gastric irritation or the ushering in of an eruptive disease; the disease, in other words, being *purely convulsive* in character."

Though not so apposite to the above reasoning, yet this observation may be made: that we have seen a child recover from excessive irritative diarrhea by the administration, through mistake, of an over-dose of laudanum sufficient to induce protracted narcotism. So also, in corroboration, the case related to me by a friend, where five grains of morphine at a dose, inadvertently used, immediately relieved a violent and seemingly unmanageable attack of dysentery. In both these instances, where a favorable result was unintentionally brought about, we would scarcely have ventured upon the means—hence the experience is the more valuable, and imparts a certain, though not very definite idea, respecting the nature of the morbid conditions in the two potents respectively.

*Experience regarding the operations for Spermatorrhea, with Lallemand's Porte-Caustique, and by a Concealed Sponge, saturated with a Solution of Nitrate of Silver. M. Trousseau's Method.*—In the second number of this Journal, Vol. V, 1850, I reported the cases of two individuals very much reduced by seminal losses, both nocturnal and diurnal, with its usual distressing effects; in the one the original cause of the disease being the presence of ascarides in the rectum. They were completely *cured* by the application of the caustic, after M. Lallemand's method, and with his instrument. Since that period I have operated seventeen times upon eight persons, and with varying success. I may say, without presenting the cases in detail, that it has in the majority proved less serviceable than I was at first induced to hope. Though the patients in some instances have not had an immediate or final end put to their seminal losses by the caustication of the prostrate gland at the points of entrance of the seminal ducts, yet they have scarcely ever failed to apply for a repetition of the means used, notwithstanding the pain resulting, and the risks inseparable from the operation; I have indeed operated on but one individual less than twice, and it was always done at his own request. I would, therefore, still advise recourse to it in cases dependent upon relaxation of the mucus membrane, and of the genito-urinary apparatus generally, attended with diurnal loss, whether it occurs with the escape of urine or accompanies the straining at stool. Iron, strychnine, and other tonics, with cold application, may, undoubtedly, also prove serviceable, and be adapted to certain cases.

But I desire, also, to call attention to other instruments as substitutes for Lallemand's. This, independently of its costliness, when made from platinum, which alone secures sufficient strength and guards against the corrosion of the metal by the caustic in contact with it, is nevertheless liable to break when the operator attempts to draw it out of the urethra after the caustication of the prostrate. This is quite like to ensue upon repeated use of the same instrument, and cases reported in the journals are not infrequent where the cup loaded with nitrate of silver has been left in the bladder, requiring for its removal other and severer measures. It has happened to myself to have the cup concealed in the body of the catheter so violently strained by the contraction of the urethra as to drop off upon the floor upon its withdrawal. The physician should never use one of these instruments made entirely of silver as they are so often sold.

To avoid all risks, I have substituted two instruments, but both undoubtedly less efficient than M. Lallemand's. One was sent to me by Prof. Ford, of Augusta, Ga., invented by himself. By means of this, a strong solution of nitrate of silver is *injected* after its introduction into the urethra upon the opening of the seminal ducts, by means of a metalic rod passing through the catheter, its point fitting in it so tightly as to act as a piston, and forcing the solution (previously introduced) through a small apperture at its distal end.

The other instrument to which I wist to refor, is constructed very much like Lallemand's, with the difference:—that the cup of his instrument to be loaded with melted nitrate of silver is supplied by a bit of sponge rolled tightly around the end of the central rod and saturated with a strong solution of nitrate of silver. This can be unmasked when it reaches the proper position, and the caustic made to come in contact with the relaxed tissues, including the mouths of the seminal vessels. And very recently, indeed, I have accidentally adopted a simple expedient, rendering the operation quite safe, which I have just put into practice. It consists in pouring a strong solution of nitrate of silver through the orifice of a hollow catheter after its introduction to the neck of the bladder, which it reaches through perforations made at the distal end, extending around an inch or so from this extremity.

I desire, also, to call attention to M. Trosseau's paper upon the treatment of seminal emissions, which was republished among the abstracts of the Journal, Vol. XI, No. 5. He there refers to the utility of introducing an instrument (which he describes) into the rectum to cause the requisite pressure upon the neighboring seminal organs—thus affording a mechanical impediment to wasting discharges. But particularly he alludes to the use of repeated small doses of a sedative, like belladonna, in those cases of spermatorrhea dependent upon morbid sensibility. To this he adds the use of *hot* baths of salt water, or warm sand applied to the perineum and loins; or even causing the patient to stand for a length of time before a fire—means much more effectual than cold in this class of cases. The reason is obvious, and I have repeatedly seen marked relief follow their employment.

*Case of Traumatic Tetanus successfully Treated with Cannabis Indica, Calomel, etc.*—Called July, 1860, to see S——, the servant of Mr. H——, aged forty-five, apparently stout and healty in person, and had previously enjoyed perfect immunity from dissease. Found him suffering from a feeling of tightness and compression about the abdomen and chest, with an inability to speak or swallow with ease. Learned that he run a nail in his foot some three weeks previously, and that, during the continuance of the pain and swelling caused thereby, he had taken a long walk, carrying a large burden upon his back.

Symptoms of *tetanus* came on gradually, and they slowly increased in severity till I was sent for. Upon close examination, noticed the contraction of the sterno mastoid, and other muscles of the neck; the muscles of the abdomen were also exceedingly tense and hard. The patient was unable to open his jaws more than a half inch apart, which symptom, together with difficulty in swallowing, continued for three weeks; during which time he was under treatment, till gradually recovery was brought about. Besides the spasms of muscles above alluded to, which varied in intensity, from time to time he suffered from sudden attacks of more violent spasms, principally affecting the muscles of the abdomen, back, neck and jaw. These often occurred with great suddenness and severity at night. He was never able to lift one foot over the other, and always walked slowly and with difficulty, as if suffering from pain and fearing to excite reflex action. A prominent symptom was excessive perspiration, principally upon the face and forehead.

As it was a case of *tetanus*, I determined to treat him with an extemporaneous prescription, fashioned after my own views, so I ordered: ℞. Calomel grs. ii; Iron, Quevennes, gr. i; Quinine gr. $\frac{1}{2}$; Opium pulv. gr. $\frac{1}{2}$, every two hours. In the intervening hour he took ten to fifteen drops, sometimes increased to thirty drops, of the Tinct, of the extract of Cannabis Indica, purchased at Messrs. Stoney & Wiltberger's. He recurred constantly to the Tinct. of the Cannabis Indica whenever the spasms became violent, seeming to feel great relief from it, and never desiring to go beyond twenty or thirty drops. The only alteration in the prescription above mentioned was in exchanging the opium in the first prescription for a half grain of morphine, and giving an occasional purge of castor oil. During a later period in the treatment he had a solution of cream of tartar and camphor water occasionally given him. On one occasion when Flemming's Tinct. Aconite was substituted for the Tinct. of Cannabis Indica, it was given up as it did not seem to prove as useful. The recovery was slow and tedious but perfect.—He took in all an enormous amount of calomel and opium, and was not salivated, nor, indeed, did it have much appreciable effect upon the secretions. The intellect all the while continued unimpaired.

*Use of cold Water (irrigation) in restraining Inflammation and preventing Heat, Pain and redness in Fractures.*—The extremely satisfactory results that I have obtained in the Marine Hospital during the past winter by the continued application of cold water to the fractured limbs, induces me to add my testimony to that of Drs. Smith, Pope, and other surgeons. It would be difficult to use terms too extravagant in describing the ease, comfort, and prevention of pain which this method of treatment secures to the patients even after double compound comminuted fractures have been sustained. During its employment the individual feels no pain; he has no fever, and he does not obviously loose flesh; he does not suffer from constitutional disturbance of any kind, nor is he conscious indeed that so serious an injury has befallen him, save when his eye is directed towards the member under treatment. At the end of the fifteenth day after entrance the patient's physical condition is just as good as on the day of his admission ; because, the limb being bathed day and night in fresh supplies of cold water, there is no pain, redness or inflammation, consequently no swelling, suppuration or wasting discharge, involving constitutional disturbance, loss of rest, emaciation, night sweats or loss of appetite. These are the usual concomitants of every other mode of treatment of fractures where the soft parts were injured.

The leg is placed in a box made of block tin, in which it fits loosely and which embraces it above the knee. This is stuffed with cloth or paper at suitable intervals to keep the leg fixed. These substances may be removed one by one and the member cleansed without disturbing its position.—At the most dependent portion of the box there is a tube passing out so far that the water falling upon the limb may escape outside of the bed. A bit of oil cloth might be used as a substitute when the box cannot be had. No straps or bandages are used. A jar containing water, with alum, (and ice if the weather is warm) is suspended above the limb by cords attached to the four posts of the bed. From the jar are depended several strips of cloth or candle wick, so directed as that the water, by capillary attraction, constantly drops upon the entire surface of the wound. This irrigation is kept up night and day for twelve to fifteen days, at which time it is advisable to discontinue it, as the action of the water becomes injurious by creating irritation of the skin. Even in the coldest weather in winter this may be kept up by covering the patient's feet with carded cotton to preserve a sufficient amount of warmth. Otherwise the temperature of the foot becomes too much lowered.

The above method has been employed in five cases of fracture of the tibia with perfectly satifactory results in every instance. In the management of wounds, recent contusions, etc., whether connected or not with injury to the bones, the use of cold water has been, in my experience, equally applicable and successful.

*Management of Chronic Ulcers.*—To secure with perfect certainty the healing of *chronic ulcers* whether situated or not on parts unfavorable to their healing, I have adopted a modification of the above plan.

It was suggested by the case of a man of scrofulous diathesis under treatment in the Marine Hospital two years ago, whose whole person was marked with old cicatrices of frequent former ulcerations. He informed me that he had been treated in the Dreadnought Hospital, near London, where they are in the habit of managing such sores by light bags made of cotton or thin cloth, somewhat resembling a miniature pillow, that will retain fluids, which are frequently soaked in *hot* water and kept applied to the ulcerated surfaces. The difference in the temperature of the water adapts this mode of treatment to this peculiar form of disease by the special influence of hot application in lessening *morbid sensibility.* In healing ulcers, I have frequently directed that the bags should be immersed in cold water, first stimulating the ulcerated surface to great activity by the previous application of strong nitric acid—or nitric acid in which bits of copper have been dissolved. The escharotic is used every two or three days, the bags soaked in hot or cold water being applied continuously. Rest is enjoined and tonics and haemetics made use of.

Fifteen cases were treated in the Hospital within the past fifteen months, and in every instance the patient has been discharged cured. After trying within the past three years every species of salve, local astringents, caustics, etc., in the attempt to come to some conclusion as to the relative value of each, and having been taught in earlier life to regard chronic ulcers as the opprobria of medicine, I now feel entirely confident that a case can hardly be so bad that it cannot be healed successfully after the method recommended. Granting the special value of *hot* applications in lessening morbid sensibility in parts extremely liable to it, I have yet thought that *cold* possess their special advantages, also, in constringing the capillaries which have been relaxed by injury to the nerves governing their due contraction—as shown by the surrounding heat and redness; therefore, some judgment may be exercised in selecting the one or the other.

J. H. Bennet says (Clinical Lectures, p. 153, "General Treatment of Exudation"): "A correct treatment, therefore, will be influenced by the stage and nature of the exudation. To prevent or diminish the extent of an exudation, we must adopt measures to overcome the dilatation of the capillaries, their distension with blood, and the attractive power, (whatever it is,) which draws the liquor sanguinis into the surrounding textures. This is accomplished—1st, by local applications of cold and astringents, which stimulate the capillaries to contraction; 2d, by soothing topical applications, such as warm fomentations, opiates, etc., which relieve the irritation of the nerves in the part." * * * * "Thus locally, *cold*, dryness and pressure check; while *heat*, *moisture*, and room for expansion, favor growth."

